# Scarification lengthening technique for the proximal pedicle medial gastrocnemius flap in the management of open leg fractures

**DOI:** 10.11604/pamj.2025.50.37.45395

**Published:** 2025-01-29

**Authors:** Ferdinand Nyankoue Mebouinz, Fabrice Stephane Arroye Betou, Freddy Bombah Mertens, Jean Paul Ndamba Engbang, Daniel Handy Eone

**Affiliations:** 1 Faculty of Medicine and Biomedical Sciences, University of Yaoundé I, Yaoundé, Cameroon,; 2 Faculty of Medicine and Pharmaceutical Sciences, University of Douala, Douala, Cameroon

**Keywords:** Flap, gastrocnemius muscle, lengthening, coverage, open fractures, leg

## Abstract

Open leg fractures frequently result in loss of substance, which can compromise the functional outcome of the lower limb. The resulting bone exposure presents a number of problems, including the need for coverage, which necessitates the utilisation of flap techniques. The medial gastrocnemius muscle flap with a proximal pedicle has an arc of rotation that restricts its use to covering the knee and the proximal epiphysis of the tibia. In this work, the authors propose a step-by-step technique for lengthening the medial gastrocnemius flap by scarification. This increases the flap's arc of rotation, compensating for the loss of bone substance and exposure of the tibia beyond its proximal epiphysis.

## Introduction

Open leg fractures are complicated by loss of substance, with exposed bone occurring in 23.5% [1]. A high risk of osteitis and septic pseudarthrosis exists, in the order of 11% and 12%, respectively [2]. Surgical management of high-energy trauma to the extremity with exposure of the fracture site requires rapid reconstruction of the soft tissue envelope to prevent infection and achieve bone healing. In the leg, where there is significant bone exposure, the loss of musculocutaneous substance requires the use of muscle flap coverage techniques [3]. Compared with fascio-cutaneous flaps, the latter have the advantage of providing a better vascularised muscle cushion, which helps to fight against infection and promote consolidation [4]. The medial gastrocnemius flap with proximal pedicle is used to cover the proximal tibia. Its classic rotation arc limits it to covering loss of substance in the knee and proximal tibia. The objective of this case study is to present the surgical procedure for lengthening the medial gastrocnemius flap by scarification, thereby increasing its arc of rotation and covering bony exposures of the tibia beyond the proximal epiphysis

## Case study

**Figure 1:**
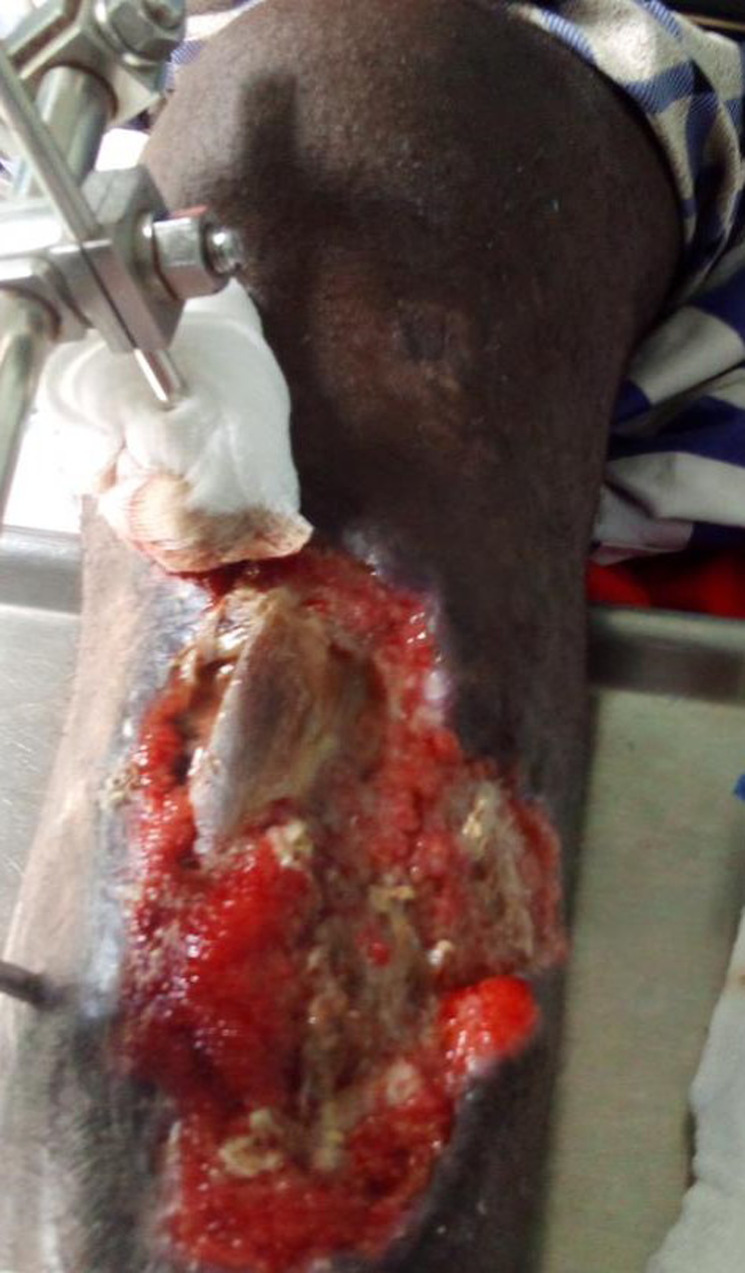
open leg fracture complicated by osteitis of the tibia and musculocutaneous damage

It is the case of a 39-year-old male non-smoker who presented with an open fracture of the proximal third of the tibia following a road traffic accident. The fracture was graded Gustilo Anderson IIIa, with soft tissue contusion graded Tscherne 3. The bruise was on the anterior medial aspect of the tibia, about 6cm by 12cm in length and extending from 4cm below the tibial tubercle to the mid-leg. The wound was debrided and external fixation was performed with an AO monoplanar tibiotibial exofixation. On the 7^th^ post-operative day, the development was characterised by a significant drop of fasciocutaneous pressure sores. Debridement during dressing revealed musculocutaneous loss with significant bone exposure of the tibia. The loss of substance was located on the anterior aspect of the leg, with a slightly oblique axis from top to bottom and from medial to lateral, opposite the junction between the upper 1/3 and the middle 1/3 of the tibia; the edges were irregular. The wound bed showed peripheral muscle excavation with central bone exposure. Approximately 6cm of the tibia was exposed with incipient superficial osteitis of the anterior cortex (Figure 1). The surgical indication was for the healing of the osteitis with the collection of samples for cytobacteriological examination, followed by a proximal pedicle flap of the medial gastrocnemius muscle. During this operation, the surgeon had to lengthen the flap due to difficulties in covering the muscle and bone without tension. After a favourable outcome with satisfactory budding, a thin skin graft was performed. The last X-ray of the leg showed bone consolidation.

### Surgical technique

The operation was performed under local spinal anaesthesia. The patient was positioned supine on a standard table. Standard asepsis was performed on the patient, consisting of washing with iodinated betadine scrub over the entire limb, followed by rinsing with 70° alcohol and then swabbing with 10% yellow iodinated betadine. Surgical draping was then performed with sterile non-woven drapes, leaving the entire left lower extremity free in the operating field.

### Debridement

The initial stage of the operation involved debridement, followed by the treatment of tibial osteitis through curettage and superficial resection of the mortified anterior tibial cortex. This resection was carried out progressively through the thickness of the cortex, over the entire height of the infected part of the bone, using an osteotome and Friedman gouge forceps. The procedure was deemed satisfactory and was stopped when a well-bleeding cortex was visible. The edges of the fracture were also curetted using a curette.

### The harvesting of the proximal gastrocnemius flap

**Figure 2:**
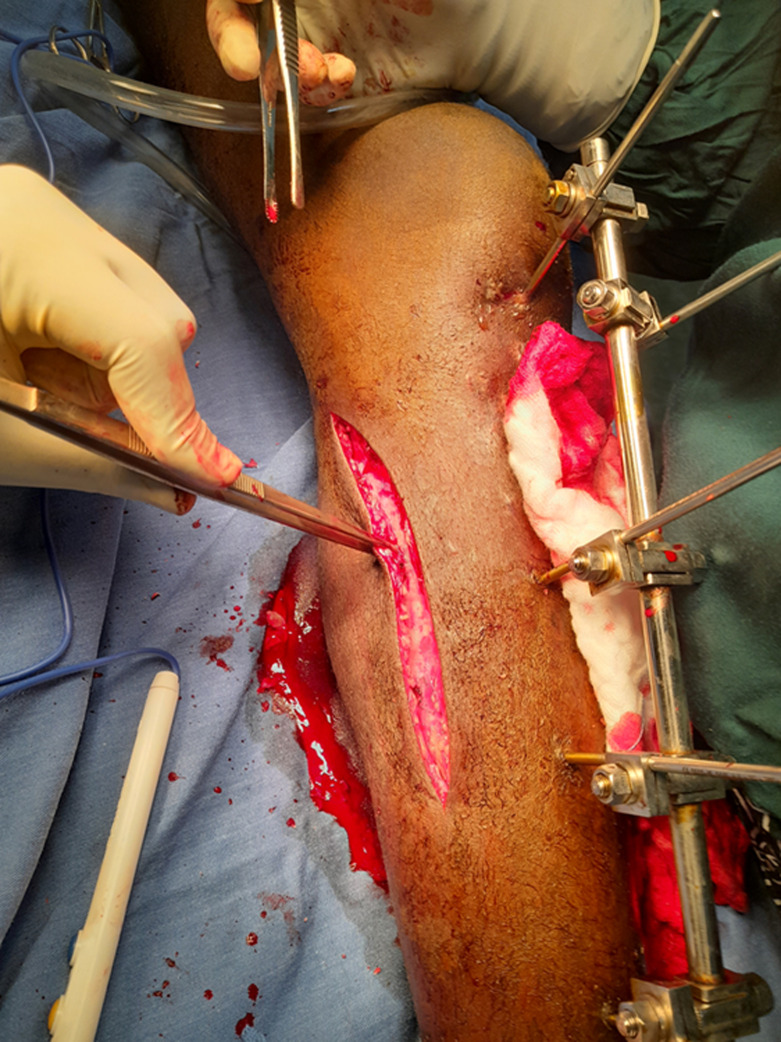
skin incision for the surgical approach to the medial gastrocnemius flap

Following debridement of the osteitis, the limb was placed in external rotation with the pelvis tilted slightly clockwise and the knee bent slightly to expose the medial aspect of the leg. The incision (Figure 2) started mid-calf, 2cm behind the posteromedial border of the tibia and curved proximally towards the popliteal fossa to avoid damage to the saphenous nerve and vein. The deep fascia was incised in a direction parallel to its fibres, in continuity with the skin, followed by a wide fasciocutaneous detachment. The deep fascia was exposed with the Metzembaum scissors up to the demarcation line of the two heads of the gastrocnemius muscle. The intermuscular plane between the soleus muscle and the medial gastrocnemius was detached with a finger after a fine incision of the fascia. The line of demarcation between the two heads of the gastrocnemius was identified on their anterior surface with the finger and they were separated, ensuring the distal neurovascular pedicle of sural flap was preserved. The distal tendon was then sectioned, after which the medial chief was lifted distally to proximally by sectioning the fascial layer separating it from the lateral chief.

### Flap lengthening, tunneling and coverage of bone exposure

**Figure 3:**
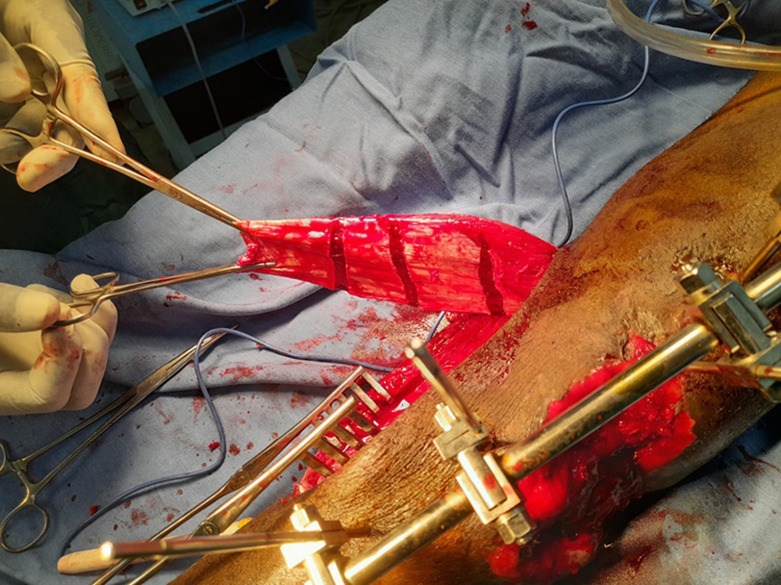
lengthening of the muscle flap by scarification of the deep fascial layer

**Figure 4:**
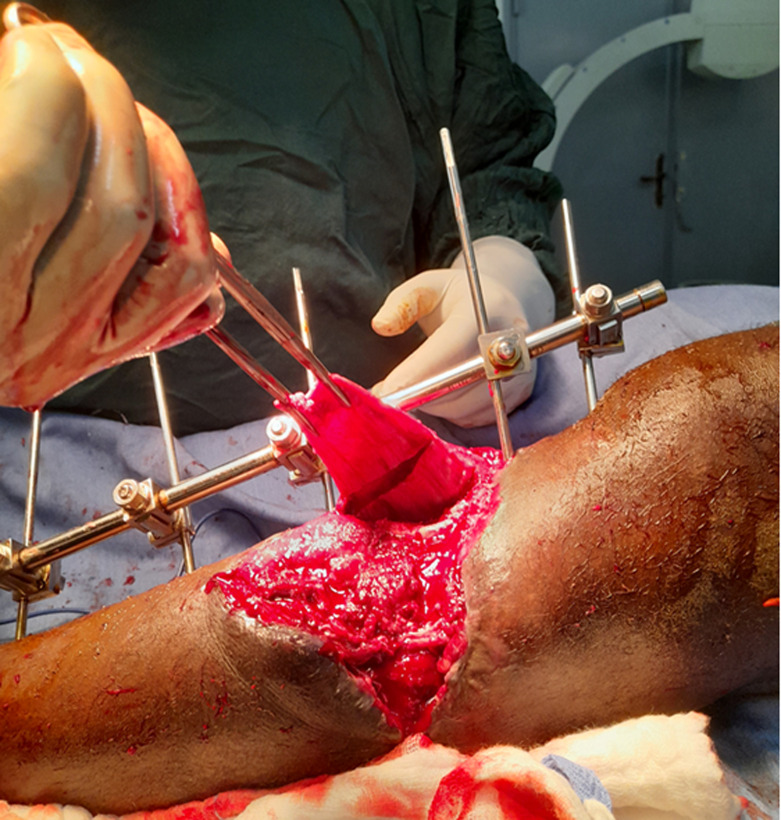
passage of the elongated flap under the tunnel

**Figure 5:**
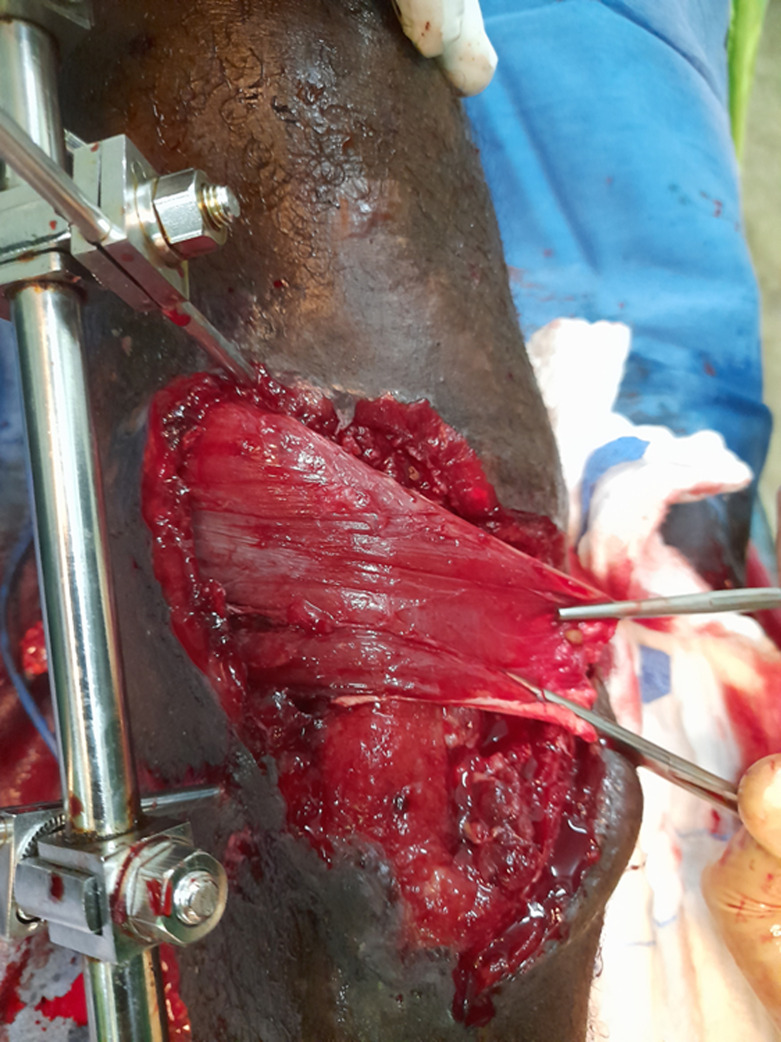
coverage of bone exposure of the tibia by the elongated flap

To ensure comprehensive coverage of the exposed tibia up to the junction of the proximal third and middle third, three transverse incisions were made in the deep fascial layer of the medial gastrocnemius head (Figure 3). A tunnel was then created under the superficial aponeurosis of the leg, taking the shortest route to the bone exposure (Figure 4). It was important to ensure that the flap was not compressed or constricted as it passed under the fascia. The elongated, tunneled flap was then fanned out over the bone exposure site, ensuring that it was covered without tension (Figure 5). The wound edges were then secured with absorbable sutures.

### Controlled healing, thin skin grafting and bone consolidation

**Figure 6:**
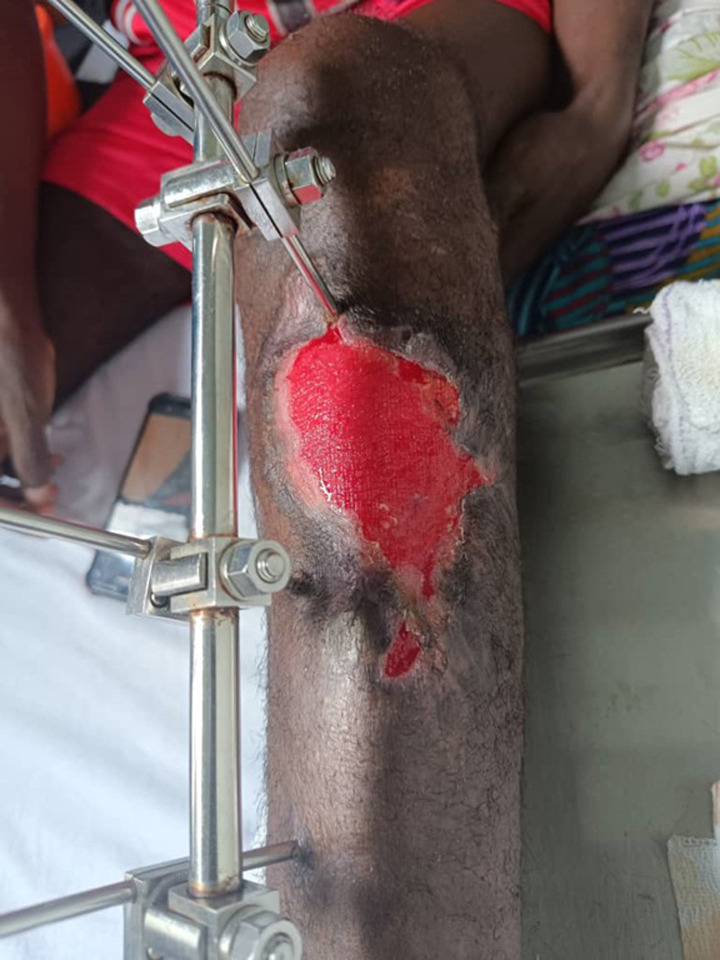
directed wound healing in the budding phase on postoperative day 28

**Figure 7:**
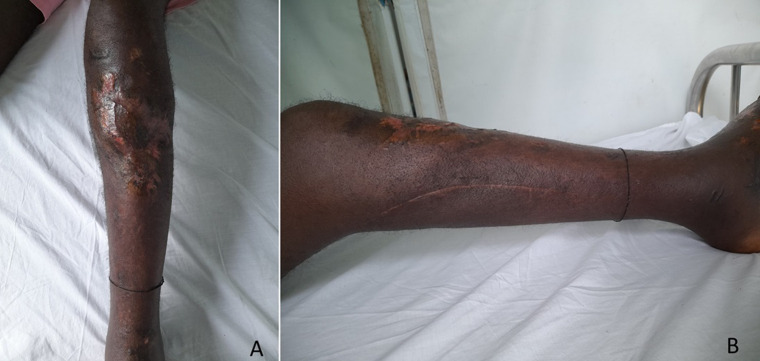
surgical scar after thin skin grafting, external fixator removed; A) anterior view of the leg; B) medial view of the leg showing good healing of the flap incision

Subsequent management involved the application of multiple oily dressings, followed by the implementation of a thin skin graft when the wound bed had become regular, red, with clean edges and an epidermal stop (Figure 6, Figure 7). The patient was admitted to the hospital for a period of five months. Radiographically, bone consolidation was achieved at four months post-operatively. At five months, the patient was permitted to bear weight on the affected limb. To date, the patient is able to walk without weight-bearing, with a slight limp and no joint limitations in the knee or ankle.

**Ethics statement:** the patient provided written informed consent for publication of the research details and clinical images.

## Discussion

This work presents a technique for lengthening the medial gastrocnemius flap and its use in the coverage of post-traumatic bone exposure of the anterior aspect of the leg at the mid-proximal junction. In the leg, the muscles of each compartment can be used to create a muscle flap for the coverage of a loss of substance or post-traumatic bone exposure. The vascular type of the muscle largely determines the method of lifting it, which may be by translation, rotation or inversion. The use of muscle flaps in the therapeutic armoury of techniques for covering loss of substance with post-traumatic bone exposure in the leg is well documented. The numerous advantages of these materials render them invaluable allies in a number of applications. These include infection control, vascular richness, malleability, and their role in the revascularization process of necrotic bone and bone grafts, among others [5]. In fact, muscle tissue is much more vascular than skin tissue. The muscle tissue exhibits a capillary density of 1,000 to 2,000 per square millimetre, in comparison to the skin, which has a density of 10 to 55 per square millimetre [6]. Furthermore, this vascularisation facilitates the consolidation of open fractures where the fracture haematoma has been evacuated by trimming. The vascularisation of the gastrocnemius muscle is provided by the sural arteries, which arise from the popliteal artery and correspond to type I of the classification system proposed by Mathes and Nahai [7]. In this type of flap, each muscle head can be mobilised individually on its own neurovascular pedicle. This results in a greater potential for rotation of the flap. Each muscle head is characterised by a dense vascular network and multiple intramuscular collaterals, which collectively reduce the risk of muscle necrosis following the section of the aponeurotic blade to lengthen it. This is of particular significance, given that the aponeurotic blade serves a supportive function and is not vascularised.

The medial gastrocnemius flap with proximal pedicle has an arc of rotation which restricts its use to the anterior aspect of the knee and the proximal epiphysis of the tibia. In the middle of the leg, coverage of bony exposures is more reliant on the distal pedicle soleus flap [8]. Nevertheless, the harvesting of this flap is less straightforward than that of the medial gastrocnemius, and there is a greater risk of vascular and nerve damage. To compensate for this limitation in coverage due to its rotational arc, several authors have described different techniques for lengthening the medial gastrocnemius, with varying gains in length and varying degrees of morbidity. The disinsertion of the medial gastrocnemius involves a meticulous dissection of the medial head down to its origin in the popliteal fossa. This technique has the capacity to extend the gastrocnemius flap by up to 6cm [9]. Nevertheless, the procedure is associated with a significant decline in knee function during ambulation. Furthermore, there is a risk of vascular damage to the pedicle, and the operation takes longer. The longitudinal division of the body of the medial gastrocnemius flap is a technique that is based solely on anatomy. Indeed, the work of authors such as Agarwal *et al*. [10], who performed cadaveric dissections of the gastrocnemius flap, indicates that it can be divided in two longitudinally. However, this lengthening technique has not been sufficiently reported in the literature to allow for an assessment of its safety. The trans-tibial transposition of the medial gastrocnemius flap, as revised by Morris *et al*. [11], involves passing the flap anteriorly to the anterior surface of the tibia through a foramen created in the metaphyseal zone of the proximal tibia anteroposteriorly. It is of paramount importance to ensure that the medial and lateral bone cortices remain intact throughout the procedure.

Although this technique offers the shortest route for the flap to cover the anterior aspect of the tibia, it is associated with a number of disadvantages. Firstly, it is quite morbid, and secondly, it presents a significant risk of metaphyseal fracture and necrosis of the flap by strangulation. The technique of muscular excision of the sural pedicle was first described by Kroll *et al*. [12]. The procedure entails the release of a proximal segment of the sural pedicle from surrounding muscle tissue, thereby creating a free flap. This procedure increases the arc of rotation of the muscle flap and reduces the risk of muscle tightness in the tunnel. One of the limitations of this approach is the difficulty in restoring venous return, which can result in venous congestion and swelling of the vein. The distal gastrocnemius flap, as described by Bashir [13], is a surgical procedure that can be used to cover the middle and distal thirds of the leg. Atchabahian subsequently identified a limitation to this technique in terms of the anatomical variability of the distal medial gastrocnemius pedicle. He found that not only was this inconsistent, but that in 23% of cases, it was not large enough or long enough to allow it to be harvested [14]. The free gastrocnemius flap described by Salibian [15] can be employed to address losses of substance in the distal part of the leg. The procedure requires the surgeon to possess advanced microsurgical grafting skills. The flap is harvested with its arterial and venous pedicles. The saphenous and sural veins are the most commonly utilized grafts.

In our case, we elected to employ the technique of transverse scarification of the deep fascial blade for lengthening purposes. This technique is relatively straightforward, faster, and less invasive than other available options. Furthermore, there is a reduced risk of vascular injury and flap necrosis due to vascular collapse of the arterial network. The decision to utilise the medial rather than the lateral gastrocnemius flap is contingent upon the greater volume of the latter and its proximity to the anteromedial aspect of the tibia, which is an area that is anatomically unprotected by muscle.

## Conclusion

The use of muscle flaps in the management of substance loss remains a viable option. In the context of open fractures, which are most likely to occur in the leg, the use of muscle flaps is of great interest in the management of complications. Furthermore, an understanding of gastrocnemius flap lengthening techniques allows for the possibility of recovery with the least possible morbidity. In the context of a rising incidence of open leg trauma, these lengthening flap recovery techniques offer solutions that improve patients' functional prognosis.
